# Differentiating Resistance from Formulation Failure: Isoniazid Instability and Poor Dissolution in Crushed Multi-Drug Paediatric Preparations

**DOI:** 10.3390/pharmaceutics18030389

**Published:** 2026-03-21

**Authors:** Halima Samsodien, Jana Winkler, Marique Aucamp, Anthony J. Garcia-Prats

**Affiliations:** 1 Pharmaceutics Discipline, School of Pharmacy, University of the Western Cape, Robert Sobukwe Road, Bellville, Cape Town 7535, South Africa; 2 Desmond Tutu TB Centre, Department of Paediatrics and Child Health, Stellenbosch University, Tygerberg, Cape Town 8000, South Africa

**Keywords:** isoniazid, paediatric formulations, supramolecular instability, drug–drug interactions, drug stability, spectroscopy, thermal analysis, dissolution

## Abstract

**Background:** Bedside manipulation of adult anti-tuberculosis tablets for paediatric dosing is common in low-resource settings, yet it can compromise drug stability. This study investigated how grinding and multi-drug co-suspension affect the supramolecular organisation, thermal stability, and dissolution of isoniazid (INH). **Methods**: INH raw, INH branded tablets (whole and ground), and multi-drug combination mixtures (MCMs) that simulate paediatric multi-drug-resistant tuberculosis (MDR-TB) regimens were assessed. Samples were analysed as solids and aqueous suspensions using hot-stage microscopy (HSM), thermogravimetric analysis (TGA), differential scanning calorimetry (DSC), Raman spectroscopy, FTIR-ATR, USP dissolution, and HPLC (LOD 0.0015 mg mL^−1^; LOQ 0.005 mg mL^−1^). **Results**: Grinding and co-mixing lowered melting points and masked typical INH events. Spectroscopy revealed the broadening and shifting of OH/NH and pyridine-ring bands, consistent with the formation of new hydrogen-bonding networks, correlative with supramolecular rearrangements. In multi-drug suspensions, INH fell below the HPLC quantification limit in both pH 1.2 and 6.8 media, despite visible residue, suggesting the formation of non-dissociable supramolecular complexes. Using a validated HPLC assay, no quantifiable INH was detected from the crushed multi-drug suspensions in either pH 1.2 or pH 6.8, whereas intact API/tablets showed measurable release. **Conclusions**: Co-suspension of INH with companion tuberculosis (TB) drugs disrupts its supramolecular integrity, leading to pre-administration degradation and a loss of quantifiable drug. Dissolution testing showed minimal INH release at pH 1.2 and none at pH 6.8, contrasting with intact tablets/API. These observations highlight that converting an immediate-release tablet into an aqueous suspension fundamentally alters its physicochemical environment and requires rational formulation design to preserve molecular stability, differentiating true resistance from formulation failure.

## 1. Introduction

Isoniazid (INH) remains foundational to Tuberculosis (TB) therapy, yet its chemical behaviour and exposure profiles can be fragile under real-world handling and patient factors. Computational and mechanistic analyses indicate that INH is prone to reaction pathways (e.g., hydrazone formation and hydrolysis/oxidation routes) that may be triggered or accelerated by the formulation environment and processing conditions [1]. Clinically, INH pharmacokinetics in children show substantial variability, raising the risk of sub-therapeutic concentrations during routine care [2]. Comparable concerns have been documented in adults, where INH pharmacokinetics measured under programmatic conditions have been linked with treatment outcomes, underscoring the clinical importance of maintaining adequate exposure [3]. Together, these strands motivate a closer examination of how bedside manipulation and multi-drug co-suspension influence INH integrity and dissolution, issues that can masquerade as microbial resistance rather than formulation-driven failure.

Paediatric tuberculosis management continues to be hampered by the scarcity of stable, child-appropriate dosage forms. In many resource-limited settings, clinical staff or caregivers crush adult tablets and prepare aqueous co-suspensions before dosing. While this offers flexibility, it introduces risks such as chemical degradation, altered dissolution, and reduced bioavailability. This is particularly problematic in children, where sub-therapeutic exposure may drive treatment failure and resistance.

Paediatric TB therapy often relies on the bedside practice of crushing adult tablets and preparing ad hoc aqueous mixtures, which can alter solid-state order, supramolecular interactions, and dissolution. Isoniazid (INH) is susceptible to hydrolysis and carbonyl-driven reactions with reducing excipients; it is also polymorphic, with crystalline forms that may interconvert under such stress. This work investigates how grinding and multi-drug co-suspension affect INH thermal responses, vibrational band signatures, and dissolution, and interprets the findings using a correlative supramolecular framework whilst acknowledging potential polymorphic contributions.

Isoniazid is central to first-line and preventive TB therapy, but it is highly formulation-dependent. When tablets are crushed and mixed with food or common vehicles, the hydrazide can react with reducing sugars and other excipients, promoting hydrazone formation, hydrolysis, and oxidative loss of the active drug [4]. These manipulation-driven pathways lower dissolution and assayable INH, making the delivered dose unpredictable and sub-therapeutic despite correct nominal dosing [4]. In practice, such formulation failure can mimic “INH resistance”, confounding clinical decisions unless the preparation is controlled or avoided [4]. Recent reviews on anti-mycobacterial drug stability underline how degradation during sample handling, storage, or formulation manipulation can compromise assay and therapeutic integrity [5]. Drug–excipient and drug–drug interactions within multi-component systems are increasingly recognised to influence crystallinity, solubility, and decomposition pathways [6].

Supramolecularly, the ordered hydrogen-bonding and π–π stacking interactions within crystalline INH underpin its stability in solid form. The disruption of these interactions by mechanical stress, an aqueous medium, or contact with other molecules can promote structural reorganisation, amorphisation, or the formation of new intermolecular complexes [7]. Such changes may reinforce accelerated degradation or hinder dissolution in manipulated formulations.

Reformulating an immediate-release tablet into an apparent immediate-release suspension is therefore not a trivial conversion. Although both dosage forms are designed for rapid drug release, their physicochemical and supramolecular environments differ substantially. In a tablet, the crystalline INH is stabilised by compression and low molecular mobility, protecting the hydrazide moiety from hydrolysis. Once dispersed in water, however, the hydrated and dynamic environment promotes hydrogen-bond competition, lattice disruption, and accelerated degradation. Designing an effective paediatric suspension thus requires deliberate control of pH, ionic strength, and excipient compatibility to maintain both rapid release and molecular stability.

Here, a systematic approach is mimicked by interrogating how the grinding and co-suspension of INH with companion TB drugs affect its supramolecular organisation, thermal stability, spectroscopic signature, and dissolution behaviour. By coupling orthogonal analytical techniques, the aim was to elucidate the molecular underpinnings of degradation and guide future paediatric formulation design that ensures immediate release without loss of chemical integrity.

## 2. Materials and Methods

### 2.1. Materials

Branded isoniazid tablets (Winthrop, Pretoria, South Africa; excipients include lactose monohydrate, maize starch, magnesium stearate) and additional brands were donated by the Desmond Tutu TB Centre (Stellenbosch University, Stellenbosch, South Africa). All samples were stored aseptically at 20 ± 2 °C, with batch numbers and expiry dates recorded. Isoniazid raw material (batch AB0600) was supplied by Sanofi, Paris, France. All samples were stored aseptically at 20 ± 2 °C, with batch numbers and expiry dates recorded.

### 2.2. Sample Preparation

For INH-only testing, a single 100 mg INH tablet was ground in a porcelain mortar and pestle for 5 min and transferred into glass poly-top vials.

For the multi-drug combination mixture (MCM), the dosing reflected that of a 4-year-old child weighing 20 kg: INH 400 mg, pyrazinamide (PZA) 800 mg (Macleods Pyrazinamide 500 SA), ethambutol (EMB) 500 mg (Sandoz Ethambutol 400 mg), ethionamide (ETH) 400 mg (Ethatyl 250 mg (Sanofi-Aventis, Paris, France)), levofloxacin (LEV) 400 mg (Austell Levofloxacin 250 mg/500 mg (Austell Labs, Johannesburg, South Africa)), and terizidone (TER) 400 mg (Terizidone Macleods 250 mg Capsules (Macleods, Mumbai, India)). Corresponding tablets/capsules were crushed/emptied and ground for 5 min, then divided as follows: (i) MCM (dry) stored as ground powder; and (ii) MCS (suspension), to which 5 mL of distilled water was added immediately before analysis. The 5 mL water volume approximates bedside manipulation practice, and suspensions were analysed within 10 min of reconstitution.

### 2.3. Thermal Analyses

Hot-stage microscopy (HSM) was performed using an Olympus microscope (Wirsam Scientific & Precision Equipment (Pty) Ltd., Johannesburg, South Africa) equipped with a Linkam SZX7 (Linkam Scientific Instruments Ltd., Salfords, UK) heating stage, 25 °C to 400 °C at a heating rate of 10 °C min^−1^. Images and interpretations around 190–200 °C correspond to melt/degradation transitions rather than physiological conditions; physiological behaviour was assessed by dissolution at 37 °C. Thermogravimetric analysis (TGA) was conducted on a PerkinElmer TGA 4000 (Shelton, CT, USA) using 3–5 mg samples, heated at 5 °C min^−1^ to 400 °C under nitrogen at 40 mL min^−1^. Differential scanning calorimetry (DSC) was conducted on a PerkinElmer DSC 8000 (Shelton, CT, USA) with integrated cooling. Samples (3–5 mg) were sealed in aluminium pans and analysed at 5 °C min^−1^ to 400 °C under nitrogen at 20 mL min^−1^.

### 2.4. Raman Spectroscopy

Spectra were acquired on an Anton Paar Cora 5700 system (Ashland, VA, USA) equipped with a 1064 nm laser. Branded tablets, ground tablets, MCM, and MCS samples were analysed, using 10 accumulations × 20 s per measurement.

### 2.5. Fourier Transform Infrared (FTIR) Spectroscopy

FTIR-ATR spectra were acquired on a PerkinElmer Spectrum 400 (Shelton, CT, USA) with an ATR accessory, 4 cm^−1^ resolution, air as background, and no hydraulic press was used owing to the ATR configuration, over 4000–650 cm^−1^. For suspension interrogation, MCS was briefly refluxed (~10 min), vacuum-filtered (Rocker 300^®^,Rocker Scientific Co., Ltd., New Taipei City, Taiwan), and scanned by ATR.

### 2.6. Dissolution Testing

Dissolution was performed using a SOTAX Smart System (USP Apparatus I, rotating basket) (SOTAX AG, Aesch, Switzerland) with 900 mL media: pH 1.2 prepared as 0.01 N HCl and pH 6.8 USP buffer, at 37 ± 0.5 °C, 100 rpm. Test units included the following: INH API in hard-gelatin capsules (size 0) filled using Cap.M.Quick™ (Star Products, Berry Creek, CA, USA) and weighed on a Mettler Toledo AJ100^®^ (Columbus, OH, USA); intact tablets; ground tablets; and MCS (5 mL). USP Apparatus I was used for both capsules and tablets to maintain consistent hydrodynamics across formulations. Samples were withdrawn at 10, 20, 30, 45, and 60 min, filtered through 0.45 µm nylon membranes (Whatman/Cytiva (Sigma-Aldrich), Marlborough, MA, USA) validated for >98% INH recovery, and replaced with pre-warmed medium.

Although some profiles exhibited an upward trajectory near 60 min, testing was limited to 60 min to comply with the USP immediate-release specification (≥80% release in 45 min) and to prevent late-phase oxidative degradation that can confound interpretation. This timeframe captures the clinically relevant dissolution phase for bedside formulations; extending beyond 60 min would not enhance mechanistic understanding and risks secondary degradation artefacts.

### 2.7. High-Performance Liquid Chromatography (HPLC)

Quantitative analysis of INH was performed using a Knauer Azura HPLC system (Berlin, Germany) equipped with a Phenomenex Kinetex C18 column (250 × 4.6 mm, 5 µm, Torrance, CA, USA). The mobile phase comprised 20 mM sodium phosphate buffer (NaH_2_PO_4_, pH 6.8)/methanol in a 70:30 *v*/*v* ratio, delivered at a flow rate of 1.0 mL/min. Detection was set at 238 nm, shown to be specific for INH without matrix interference via blank matrix scans; the injection volume was set at 10 µL. Calibration was linear over the range 0.005–72.00 mg/mL (r^2^ = 0.9997). The method was validated for specificity, linearity (0.005–72.00 mg mL^−1^; r^2^ = 0.9997), accuracy, precision (intra-/inter-day %RSD < 2%), and LOD/LOQ (LOD 0.0015 mg mL^−1^; LOQ 0.005 mg mL^−1^) in accordance with ICH Q2(R2). A single INH-selective method was intentionally applied across matrices because the study aimed to quantify INH rather than multiple analytes. 

### 2.8. Statistical Analysis

All experiments were performed in triplicate. Data are expressed as mean ± standard deviation (SD). One-way ANOVA followed by Tukey’s post hoc test was applied using GraphPad Prism v8.0 (GraphPad Software, San Diego, CA, USA); *p* < 0.05 was considered statistically significant.

## 3. Results

### 3.1. Thermal Behaviour

**Figure 1:**
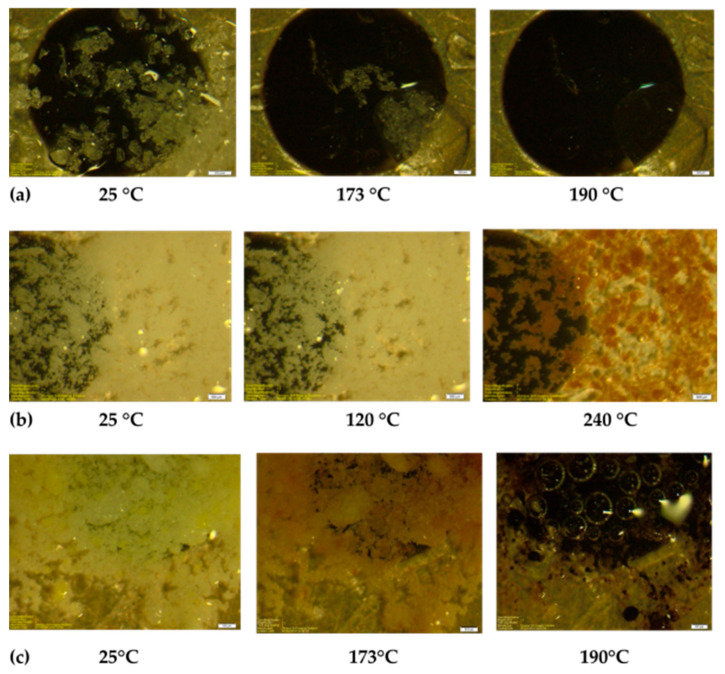
Hot-stage microscopy (HSM) micrographs (scale bar = 500 μm) showing changes in the physical appearance of (**a**) INH API, (**b**) ground INH-branded tablet, and (**c**) multi-drug combination mixture (MCM) as a function of temperature under a constant heating rate.

HSM (Figure 1) showed (a) the INH API melted sharply between ~173 and 175 °C, consistent with the literature values and (b) ground branded INH tablets softened earlier (~120 °C) and exhibited progressive darkening between 195 and 240 °C, whereas (c) MCM began melting between ~170 and 175 °C, with bubbling and matrix collapse by 190 °C. These depressed melting onsets reflect a breakdown of INH’s native supramolecular lattice due to mechanical stress and interstitial incorporation of other molecules.

**Figure 2:**
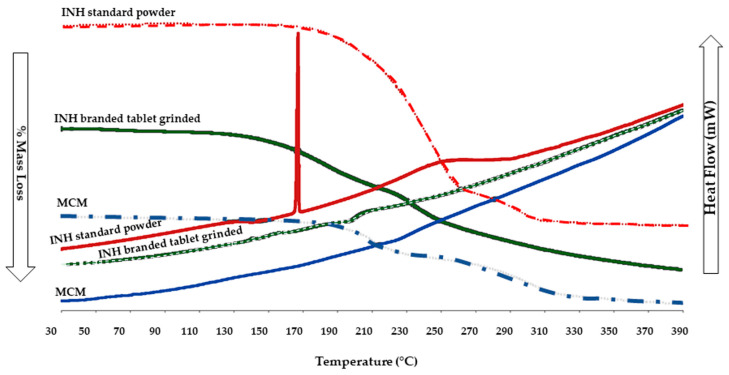
TGA curves displaying % mass loss and DSC patterns displaying heat flow in mW as a function of time, INH API (red and dotted red line), INH-branded tablet ground (green and dotted green line), and MCM (blue and light blue line).

TGA curves showed a multi-stage % mass loss; the onset temperatures followed the following order: INH API (181.4 °C) > ground tablet (173.3 °C) > MCM (156.9 °C). The progressive decline suggests increasing heterogeneity and destabilisation of molecular interactions (Figure 2).

The DSC curve (Figure 2) showed the INH API exhibiting a clean endotherm (~171.6 °C) and a broad event at 240–270 °C. The ground branded tablet displayed attenuated, shifted peaks (~146–170 °C) and an exotherm (~210 °C). MCM lacked resolvable transitions, consistent with amorphous or overlapping supramolecular domains. Overall, thermal blurring indicates a loss of long-range order and the emergence of new interactions—hallmarks of a disrupted supramolecular structure.

Grinding and co-suspension lowered the melting temperature and broadened transitions, signifying reduced crystallinity and enhanced molecular disorder. These findings suggest supramolecular disruption and possible polymorphic transformation.

### 3.2. Raman Spectroscopy Band Assignments

**Figure 3:**
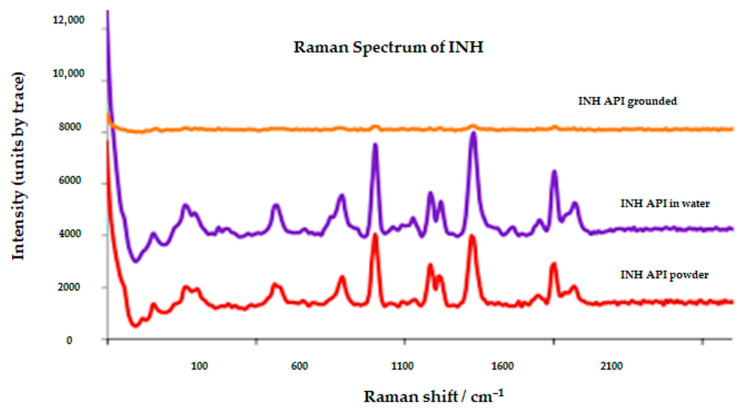
Raman spectra of INH API, INH API in water, and INH tablet ground spectra.

The Raman spectra and band assignments are shown in Figure 3 and Table 1. The INH API spectrum featured sharp bands assignable to pyridine, amide, and ring modes. After grinding and aqueous suspension, bands in the OH/NH region broadened, and the pyridine-ring peaks weakened or shifted. These spectral alterations imply the formation of new hydrogen-bond networks between INH molecules and excipients or co-drugs, replacing the original intramolecular and intermolecular interactions. Such band broadening reflects competitive hydrogen bonding between INH and excipients and supports the thermal data.

### 3.3. FTIR Spectroscopy and Molecular Interaction

**Figure 4:**
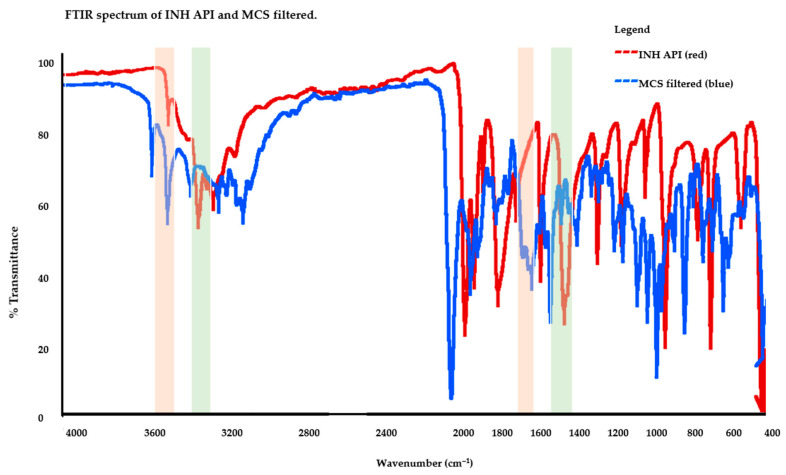
FTIR characteristic frequency bands of INH API and MCS filtered. The pink-shaded box highlights the NH/OH and C–H stretching region (≈3600–2500 cm^−1^), and the green-shaded box highlights the carbonyl/pyridine/amide fingerprint region (≈1800–1400 cm^−1^), as summarised in Table 2.

The FTIR band assignments and spectra are shown in Table 2 and Figure 4. The ν(C=O) band of INH, observed at 1662.77 cm^−1^ in the API, was retained in the MCS filtered sample but shifted to 1665.08 cm^−1^, indicating preservation of the hydrazide carbonyl functionality within an altered molecular environment. Such shifts in the amide I region are highly sensitive to changes in hydrogen-bond strength and geometry, particularly involving the hydrazide –NH and carbonyl oxygen. Mechanical grinding and subsequent aqueous exposure are known to disrupt the native crystal lattice, facilitating reorganisation of intermolecular hydrogen bonds. In the presence of companion drugs and excipients, competitive hydrogen-bond donors and acceptors can further perturb the local electronic environment of the carbonyl group. The emergence of additional or broadened carbonyl features in the ground tablet supports the formation of multiple carbonyl environments rather than simple physical mixing. While FTIR alone cannot unambiguously identify reaction products, the observed carbonyl shifts are consistent with incipient chemical transformation, such as hydrazone-type association or strongly bound supramolecular complexes. Importantly, retention of the carbonyl band rules out complete hydrolytic cleavage, instead suggesting partial structural modification or association. These alterations would be expected to reduce molecular mobility and solvation efficiency, contributing to poor dissolution. Thus, the carbonyl band behaviour provides a critical spectroscopic link between solid-state disruption and functional loss of bioavailable INH. The full set of supplementary FTIR data is provided in the Supplementary Materials (Figures S1–S10), comprising full spectra for the ground INH-branded tablet, mixtures with individual ground anti-TB drugs (ETHAM, ETHIO, PYR, LEV, TER), and the corresponding filtered fractions.

### 3.4. Dissolution and HPLC Assay of API Release

**Figure 5:**
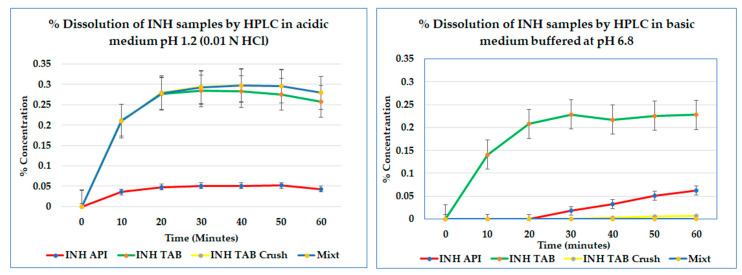
Dissolution profiles of INH API, INH-branded tablet, INH-branded crushed tablet, and mixture at pH 1.2 and pH 6.8. Note: The INH-branded crushed tablet profile (yellow) is near baseline in the left panel and therefore overlaps the x-axis, which makes the line appear absent.

In Figure 5, the dissolution results are presented. At pH 1.2 (0.01 N HCl), the INH API and tablets dissolved as expected. However, in the multi-drug suspension (MCS), visible residue persisted, and INH was below quantification by HPLC in both acidic and neutral media. The inability to quantify INH despite dissolution conditions suggests the formation of undissociable supramolecular assemblies—‘insoluble’ hydrogen-bonded complexes or drug–excipient/co-drug adducts that fail to release free INH into solution. At pH 6.8, the instability of INH is exacerbated, further reducing the recoverable drug. This behaviour aligns with the supramolecular disruption observed in spectroscopic and thermal analyses. The low recovery is attributed to insoluble supramolecular complex formation and/or matrix entrapment.

Profiles did not reach a plateau, and extension beyond 60 min was not performed, as oxidative degradation would confound interpretation. Furthermore, the HPLC assay confirmed the selectivity for INH at 238 nm and linearity (r^2^ = 0.9997). LOD 0.0015 mg mL^−1^ and LOQ 0.005 mg mL^−1^ were established. No interfering peaks were observed, verifying the method specificity.

## 4. Discussion

### 4.1. Supramolecular Disruption Drives Instability

The combined thermal, spectroscopic, and dissolution data converge to suggest that supramolecular disruption is a central mechanism of INH instability in manipulated formulations. Mechanical grinding fractures the hydrogen-bond and π–π stacking network, converting regions of the lattice into defects or amorphous domains. The introduction of water and co-drug molecules enables competitive hydrogen bonding, where INH molecules form new bonds with the surrounding compounds, thereby further undermining their original lattice cohesion. The observed spectral shifts in OH/NH and pyridine bands, and the suppression of thermal transitions, support this interpretation.

This destabilisation rationalises why INH in multi-drug suspension becomes non-quantifiable in dissolution/HPLC: INH molecules are locked into insoluble complexes or cross-linked networks, resistant to solvation. In effect, the drug loses its molecular mobility and cannot desorb into solution.

These observations substantiate supramolecular disruption as a correlative, rather than in situ, phenomenon and align with the known polymorphic nature of INH, where stress-induced conversion between crystalline forms can overlap with molecular-level reorganisation.

The thermal behaviour mirrored the spectroscopic changes: samples exhibiting the largest OH/NH band broadening also displayed the lowest onset of melting, consistent with weakened intermolecular cohesion. Such changes arise from mechanical stress and drug–excipient interactions that destabilise the hydrogen-bond network, producing metastable or amorphous states.

When co-suspended with companion drugs, additional competitive hydrogen bonds form, leading to a heterogeneous matrix in which INH molecules are partially immobilised or complexed, reducing effective dissolution and assayable content.

The dissolution profiles provide a practical manifestation of these molecular events. While intact tablets and API met the pharmacopeial specification (≥80% release within 45 min), both the ground and co-suspended mixtures showed markedly slower and incomplete release. The slight upward trajectory of the tablet curves near 60 min represents the normal asymptotic approach to equilibrium under sink conditions rather than incomplete disintegration. Testing was purposefully limited to 60 min to conform with the USP immediate-release specification and to avoid artefacts from post-release oxidative degradation, which becomes significant beyond that window. This timeframe captures the clinically relevant dissolution phase of immediate-release dosage forms; extending beyond it would introduce chemical noise without a mechanistic benefit.

Although the INH tablet is classified as immediate-release, conversion into an aqueous “immediate-release suspension” fundamentally changes the physicochemical context. In the compressed solid, the crystalline lattice and low molecular mobility protect the hydrazide moiety from hydrolysis and oxidation. Once dispersed in water and mixed with other drugs or excipients, however, INH encounters a highly dynamic hydrogen-bonding environment that promotes supramolecular reorganisation, polymorphic disturbance, and accelerated degradation. Hence, reformulating an immediate-release tablet into a liquid form demands rational vehicle design rather than mechanical dispersion.

Buffer capacity, antioxidant inclusion, redox control, and excipient compatibility must all be engineered to ensure that rapid dissolution does not compromise chemical integrity. The present findings underscore that pharmaco-technical equivalence between solid and liquid immediate-release systems cannot be assumed, particularly for chemically labile APIs such as INH, where observed dissolution may reflect degradation kinetics rather than true release behaviour.

**Figure 6:**
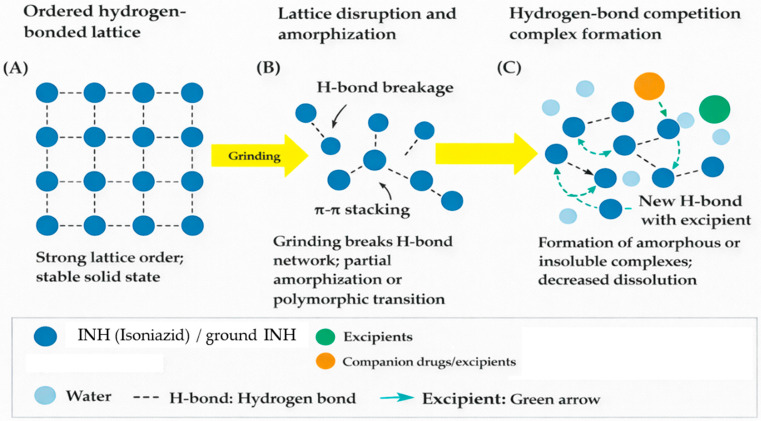
Schematic diagram summarising the proposed supramolecular pathways for (**A**) a stable crystalline lattice sustained by hydrogen bonds and π–π stacking; (**B**) partial amorphisation after grinding; and (**C**) formation of new intermolecular hydrogen bonds with co-suspended excipients or companion drugs, resulting in amorphous or insoluble complexes and diminished dissolution.

Although the FDA product label reports safe drug–drug interactions between INH and most agents studied, the current investigation focuses exclusively on pre-administration physicochemical instability under bedside conditions, not systemic pharmacology or toxicity. Figure 6 schematically summarises the proposed supramolecular pathway, with (A) a stable crystalline lattice sustained by hydrogen bonds and π–π stacking, (B) partial amorphisation after grinding, and (C) the formation of new intermolecular hydrogen bonds with co-suspended excipients or drugs, resulting in amorphous or insoluble complexes and diminished dissolution.

### 4.2. Relationship to the Drug–Excipient Interaction Literature

Our observations echo broader findings in drug–excipient compatibility research: excipient interactions can perturb crystallinity, catalyse degradation, or retard dissolution via microenvironmental effects [6]. In solid dosage design, supramolecular insights have driven the innovation of stabilising excipient systems (e.g., tailored polymer–drug hydrogen-bond interplay) that preserve the molecular order [8]. The field of supramolecular drug delivery likewise emphasises that dynamic hydrogen-bond networks and host–guest interactions underpin stability and release behaviours [7].

INH-specific work also underscores the sensitivity of its structural environment. For example, co-crystal engineering and ionic liquid strategies for INH aim to stabilise hydrogen-bonding motifs and resist degradation [9], while studies on the accelerated stability of INH polymorphs emphasise how external micro-environment changes (humidity, molecular perturbation) trigger transitions [10].

### 4.3. Practical Implications for Paediatric Formulation

These insights have immediate formulation implications: paediatric products must be engineered to preserve the molecular order and avoid undesirable intermolecular interactions. Strategies may include the following:

- Minimising aqueous residence time (e.g., rapid dispersion, immediate dosing).
- Designing excipients that preferentially support INH’s native hydrogen-bond network, not compete with it.
- Avoiding co-suspension of chemically interacting TB drugs, or spatially segregating them.
- Exploring stabilising co-crystals, host–guest inclusion structures, or ionic liquid systems for INH that resist supramolecular collapse.

Extemporaneous INH suspensions prepared at the bedside can be chemically unstable and content-variable, making the delivered dose unpredictable in practice [11]. Such instability plausibly yields poorly soluble/undissociable species that fail standard dissolution—clinically mimicking “INH resistance” rather than revealing true microbial non-susceptibility [11]. By triangulating thermal, spectroscopic, and dissolution data, this study operationalises that risk and defines formulation-control levers to prevent misclassification and exposure failure in children [11].

Critically, the supramolecular framework helps explain variabilities in dosing performance and underscores the risk of ad hoc manipulation in paediatric TB regimens.

### 4.4. Clinical/Pharmaceutical Significance

Dissolution provides the in vitro proxy for oral bioavailability. The multi-drug suspension prepared by crushing and co-mixing (MCS) failed to release measurable INH within 60 min and did not meet pharmacopoeial performance expectations for INH, whereas intact tablets/API performed substantially better. These findings are concordant with FTIR/Raman (perturbed NH/OH and pyridine bands) and thermal data (earlier mass loss, altered endotherms), supporting pre-administration degradation and drug–excipient/co-drug interactions. Clinically, the low plasma INH observed in children given crushed multi-drug slurries may reflect formulation failure, not microbial resistance, risking misclassification as “INH resistance”. Practical implications include the following: avoiding co-ground multi-drug slurries; administering drugs separately where crushing is unavoidable; minimising hold times and using acidic vehicles when appropriate; and prioritising child-appropriate, dispersible formulations. Dissolution therefore operationalises the mechanistic data and differentiates true resistance from formulation failure.

### 4.5. Clinical Evidence Supporting the Mechanism

Several clinical datasets are consistent with our bench results. In children treated for tuberculosis, sub-therapeutic INH exposures have been repeatedly documented [2]. In a predominantly HIV-infected adult cohort, INH pharmacokinetics were linked to treatment outcomes, highlighting the clinical importance of adequate exposure [3]. Programmatic paediatric experience with high-dose INH shows feasibility and potential benefit, but also notes lower observed INH concentrations when drugs are crushed and co-mixed, consistent with our finding that the multi-drug suspension (MCS) releases little measurable INH [12,13]. Contemporary guidance recognises circumstances where higher INH doses may be warranted [14,15,16], implicitly acknowledging exposure challenges [4,5].

Taken together, these clinical data reinforce our central message: low measured INH levels in patients given crushed multi-drug mixtures may reflect formulation failure rather than microbial resistance. This interpretation aligns with our spectroscopy, thermal analysis, and dissolution results showing pre-administration degradation and impaired release. Clinically, this argues for (i) avoiding co-ground multi-drug slurries; (ii) administering crushed drugs separately (minimal lag time before dosing); (iii) using acidic vehicles when appropriate; and (iv) prioritising child-appropriate dispersible or liquid formulations to ensure reliable exposure.

## 5. Conclusions

Crushing and co-suspending INH with companion TB drugs significantly disrupts its supramolecular architecture, triggering lower thermal stability, spectral perturbations, and a loss of quantifiable dissolution recovery. These findings connect molecular-level interaction phenomena with clinically relevant drug instability and support the urgent development of paediatric formulations designed for molecular integrity and compatibility.

Integrating spectroscopy, thermal analysis, and dissolution, this study shows that poor INH exposure from crushed multi-drug preparations arises from preparation-induced instability and impaired release. These results caution against routine slurry preparation and support the use of child-friendly formulations and acid-stable handling. Most importantly, they differentiate true resistance from formulation failure.

Real-world clinical observations of low INH exposure and variable outcomes with crushed regimens [2,3,12] are mechanistically explained by our data showing the structural perturbation and poor dissolution of INH after co-mixing. These results provide a practical framework for differentiating true resistance from formulation failure, supporting immediate adjustments in paediatric dosing practices and formulation choice [4,5].

## Figures and Tables

**Table 1:** Raman functional groups identified from INH API, INH API in water, and INH tablet ground spectra.

INH API (cm^−1^)	Functional Group	INH API in Water (cm^−1^)	Functional Group	INH Tablet Ground (cm^−1^)	Functional Group
3500–3220	ν(N–H) stretching (hydrazide NH/NH_2_); H-bond sensitive	3500–3220	ν(N–H) stretching broadened by hydration/H-bonding	3500–3200	ν(N–H)/ν(O–H) region (INH + moisture/excipients); broadened
3100–3000	ν(C–H) aromatic (pyridine)	3100–3000	ν(C–H) aromatic (pyridine)	3100–3000	ν(C–H) aromatic (INH)
1700–1600	ν(C=O) (amide) with ring ν(C=N)/ν(C=C) contributions	1700–1600	ν(C=O) + ring ν(C=N)/ν(C=C); shifts/broadening indicate new H-bonds	1700–1600	ν(C=O) + pyridine ring stretches (INH); intensity changes after grinding
1625–1430	Pyridine ring stretching/deformation modes (C=N, C=C)	1625–1430	Pyridine ring stretching/deformation modes (C=N, C=C)	1625–1430	Pyridine ring stretching/deformation (INH) ± overlap with excipients
1345–1265	ν(C–N) stretching (hydrazide/amide)	1345–1265	ν(C–N) stretching (hydrazide/amide)	1470–1380	δ(CH2/CH_3_) aliphatic bending (tablet excipients)
~1000	Ring breathing/ deformation around ~1000 cm^−1^	~1000	Ring breathing/deformation (may weaken/shift in hydrated state)	1100–900	Ring/C–N–N related modes (INH) ± excipient overlap
200–50	Lattice/solid-state phonon modes (crystalline)	200–50	Lattice modes reduced/altered if partial amorphisation occurs	200–50	Lattice/solid-state modes (INH + excipients); sensitive to grinding

**Table 2:** FTIR characteristic frequency bands of the INH API, INH-branded ground tablet, and MCS filtered.

Compound	Experimental Frequency (cm^−1^)	Standard Frequency (cm^−1^)	Functional Groups
INH API	3303.60	3400–3300	ν(N–H) stretching of hydrazide/primary amine; hydrogen-bond sensitive (broadening expected with H-bonding/water)
	3105.34	3100–3000	ν(C–H) aromatic (pyridine ring stretch region)
	1662.77	1640–1690	ν(C=O) amide I (hydrazide carbonyl); highly sensitive to H-bonding/chemical transformation
	1602.82	1400–1600	ν(C=N)/ν(C=C) ring stretching (pyridine skeletal vibration; can overlap with amide II region)
INH-branded tablet grounded	3412.38	3400–3300	ν(N–H) stretching broadened/shifted; consistent with stronger H-bonding and/or additional OH contribution (moisture/excipients)
	3154.54	2850–3000	ν(C–H) stretching (mixed): aromatic/weakly aliphatic contributions; likely excipient-associated envelope enhancement
	2972.10	2500–3000	ν(C–H) aliphatic (excipients; e.g., starch/lactose-associated CH envelope)
	1700.00	1670–1820	ν(C=O) shifted/upshifted carbonyl: suggests new/altered carbonyl environment (interaction/adduct formation or excipient-associated carbonyl contribution)
	1644.37	1648–1638	ν(C=O)/δ(N–H) coupled region (amide I/amide II overlap; altered H-bonding network)
MCS filtered	3108.30	3100–3000	ν(C–H) aromatic retained (pyridine C–H); modest shift consistent with changed microenvironment
	1665.08	1685–1666	ν(C=O) carbonyl retained but shifted; consistent with altered H-bonding and/or new carbonyl-containing species
	1602.83	1620–1610	ν(C=N)/ν(C=C) ring stretching shifted relative to standard; consistent with pyridine ring environment perturbation

## Data Availability

Further data are available from the corresponding author upon reasonable request.

## Supplementary Materials

The following supporting information can be downloaded at https://www.mdpi.com/article/10.3390/pharmaceutics18030389/s1, Full FTIR spectra of ground INH-branded tablet, mixtures with individual ground anti-TB drugs (e.g., ETHAM, ETHIO, PYR, LEV, TER), and the corresponding filtered fractions.
