# Supplementary material for: Differentiating Resistance from Formulation Failure: Isoniazid Instability and Poor Dissolution in Crushed Multi-Drug Paediatric Preparations
Source: Pharmaceutics. 2026 Mar 21;18(3):389. doi: 10.3390/pharmaceutics18030389 (PMC13030506; doi:10.3390/pharmaceutics18030389)
Supplement: Supplementary file 1 [file pharmaceutics-18-00389-s001.zip › pharmaceutics-4041807-supplementary.pdf]

SUPPLEMENTARY MATERIAL

Differentiating Resistance from Formulation Failure: Isoniazid Instability and Poor Dissolution in Crushed Multi-Drug Paediatric Preparations

Halima Samsodien[1]*, Jana Winkler[2], Marique Aucamp[1] and Anthony J Garcia-Prats[2]

[1]Pharmaceutics Discipline, School of Pharmacy, University of the Western Cape, Robert Sobukwe Road, Bellville, 7535, Cape Town, South Africa.

[2]Desmond Tutu TB Centre, Department of Paediatrics and Child Health, Faculty of Medicine and Health Sciences, Stellenbosch University, Tygerberg, South Africa
*Correspondence: hsamsodien@uwc.ac.za

Figures S1–S10. Full FTIR spectra of ground INH-branded tablet, mixtures with individual ground anti-TB drugs (e.g., ETHAM, ETHIO, PYR, LEV, TER), and the corresponding filtered fractions.

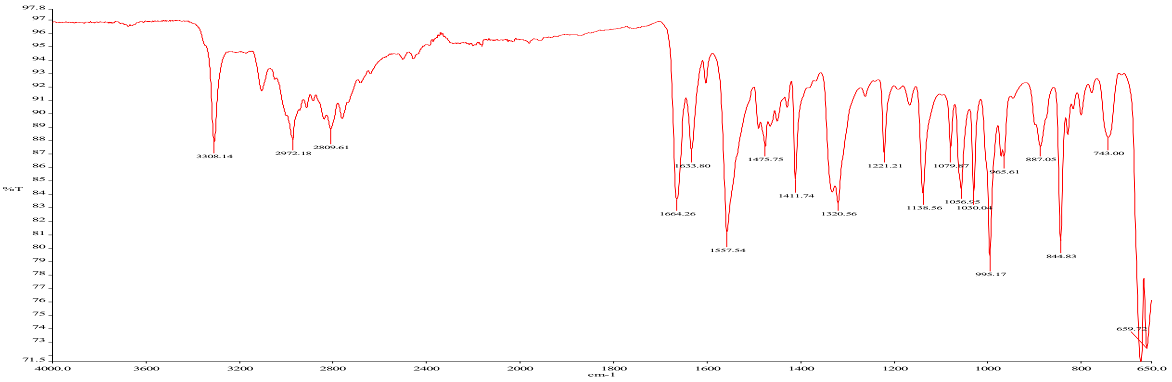

Figure S1. FTIR spectrum of ground INH mixed with ETHAM.

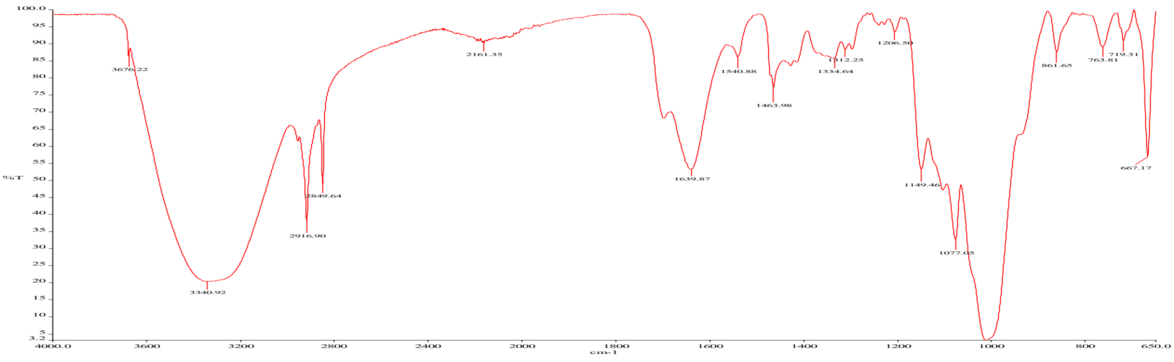

Figure S2. FTIR spectrum of ground INH mixed with ETHAM in a suspension.

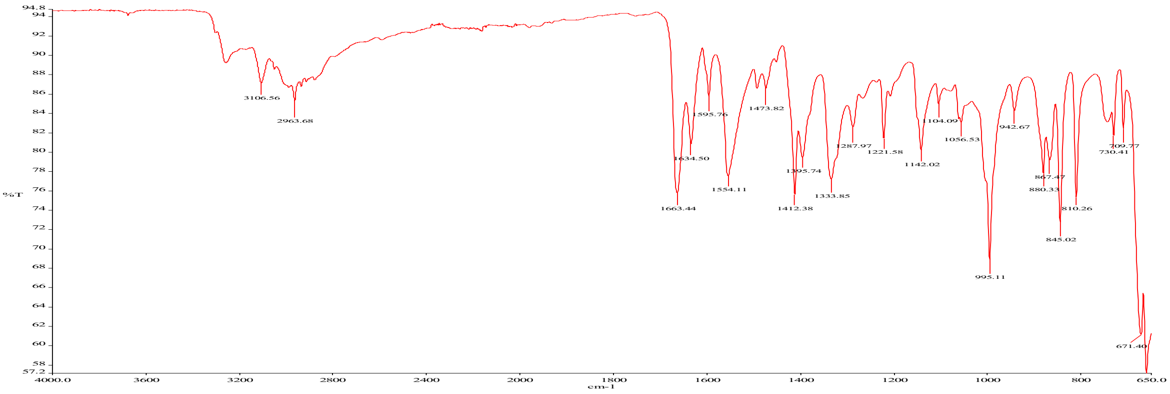

Figure S3. FTIR spectrum of ground INH mixed with ETHIO.

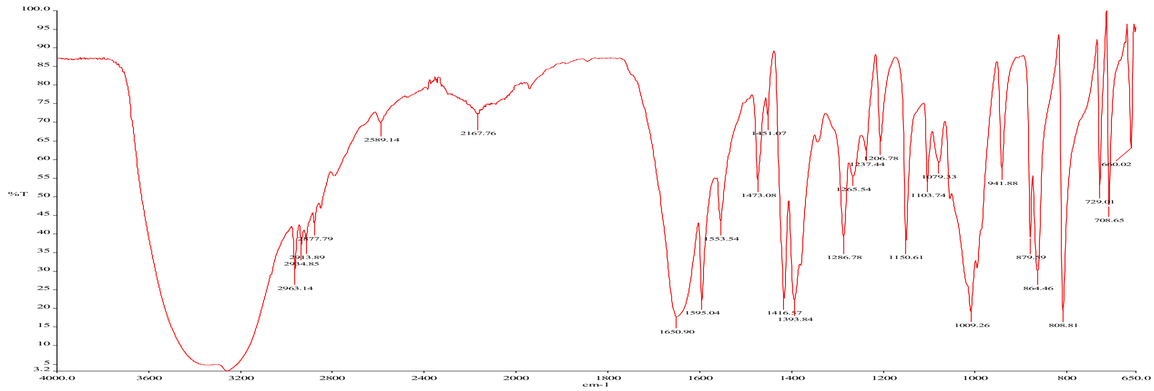

Figure S4. FTIR spectrum of ground INH mixed with ETHIO in a suspension.

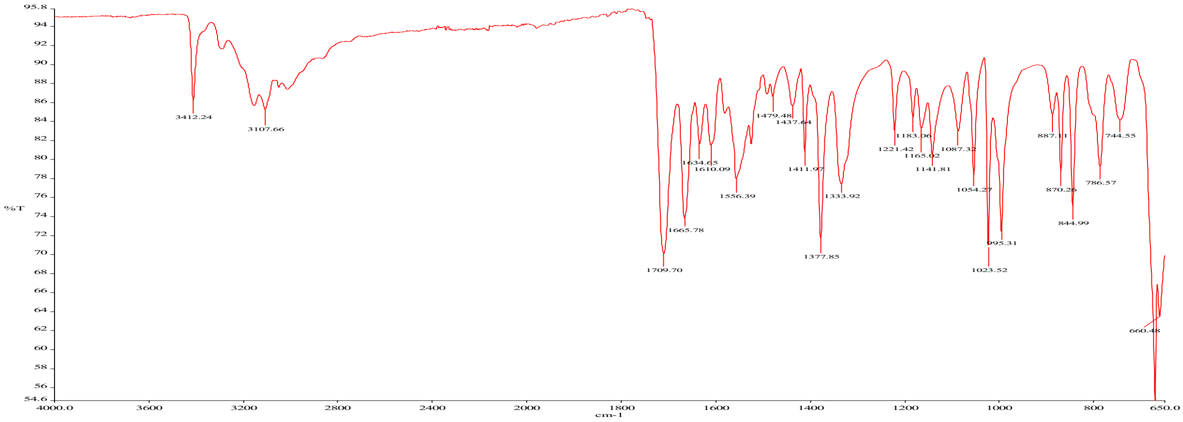

Figure S5. FTIR spectrum of ground INH mixed with PYR.

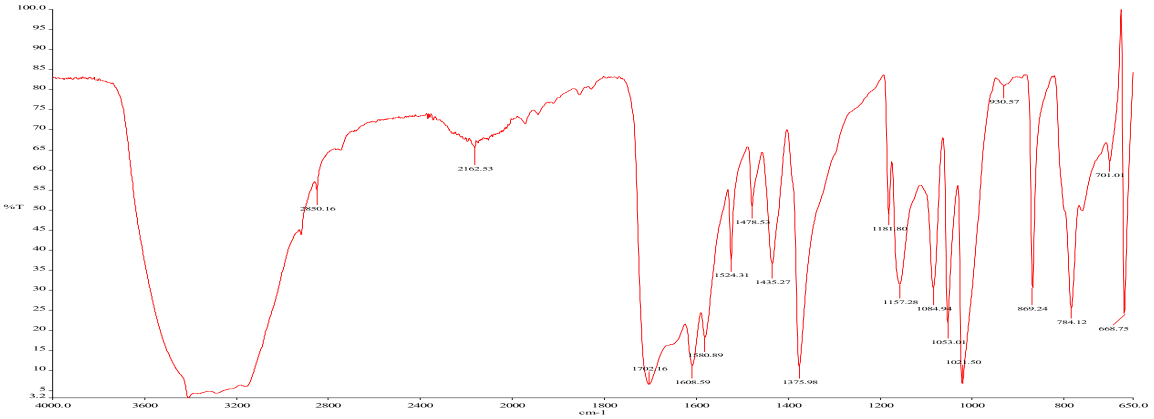

Figure S6. FTIR spectrum of ground INH mixed with pyrazinamide in a suspension.

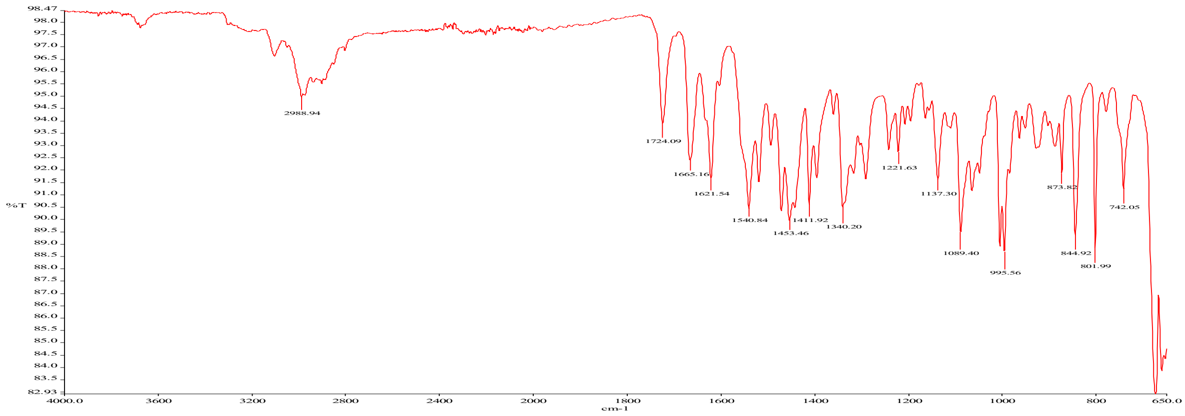

Figure S7. FTIR spectrum of ground INH mixed with LEV.

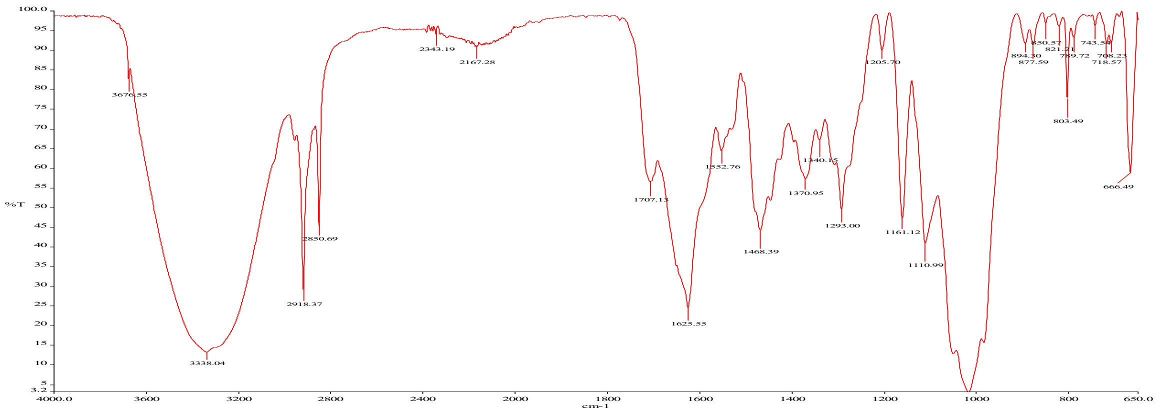

Figure S8. FTIR spectrum of ground INH mixed with LEV in a suspension.

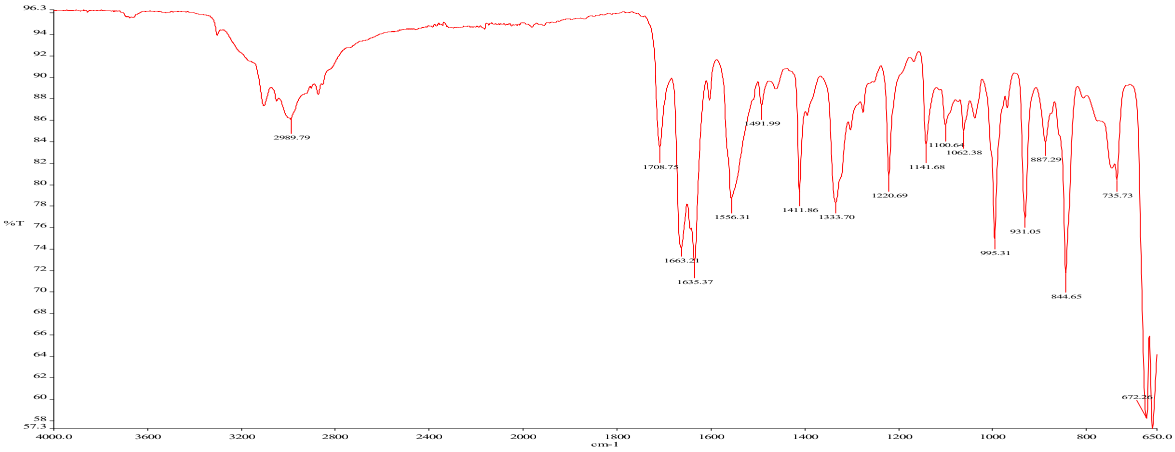

Figure S9. FTIR spectrum of ground INH mixed with TER.

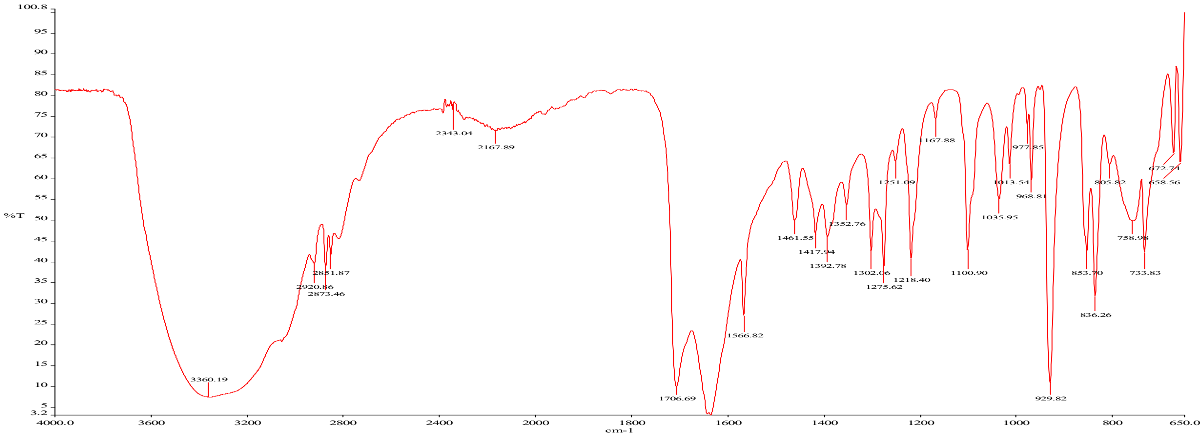

Figure S10. FTIR spectrum of ground INH mixed with TER in a suspension.
